# Supplementary figures and images for: Identification and validation of hub genes involved in foam cell formation and atherosclerosis development via bioinformatics
Source: PeerJ. 2023 Oct 3;11:e16122. doi: 10.7717/peerj.16122 (PMC10557941; doi:10.7717/peerj.16122)

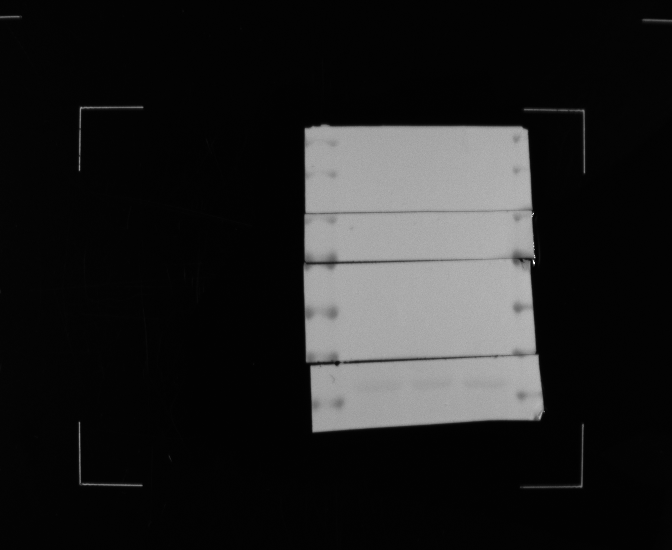

Supplement: Supplemental Information 3 [file peerj-11-16122-s003.zip › WB supplement File/First repeat/CSF1R/ALLí¬í¬1_20230509_160046_00.00.846_8bit.tif]

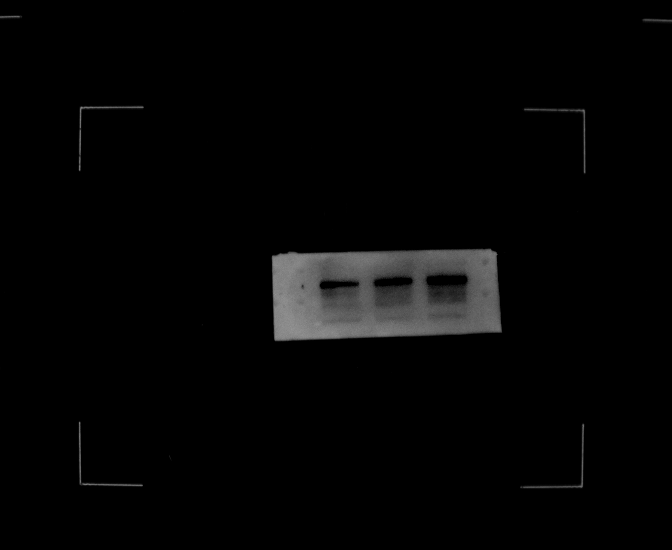

Supplement: Supplemental Information 3 [file peerj-11-16122-s003.zip › WB supplement File/First repeat/CSF1R/CS_20230509_155311_00.55.351_24bit(1).tif]

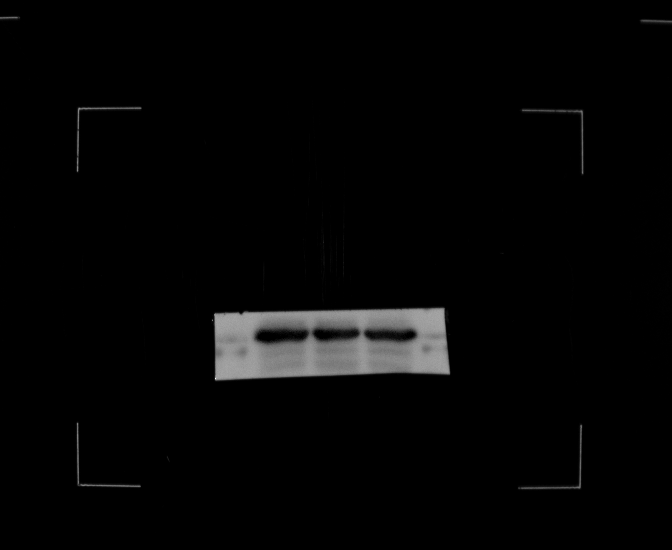

Supplement: Supplemental Information 3 [file peerj-11-16122-s003.zip › WB supplement File/First repeat/CSF1R/GAPDH.tif]

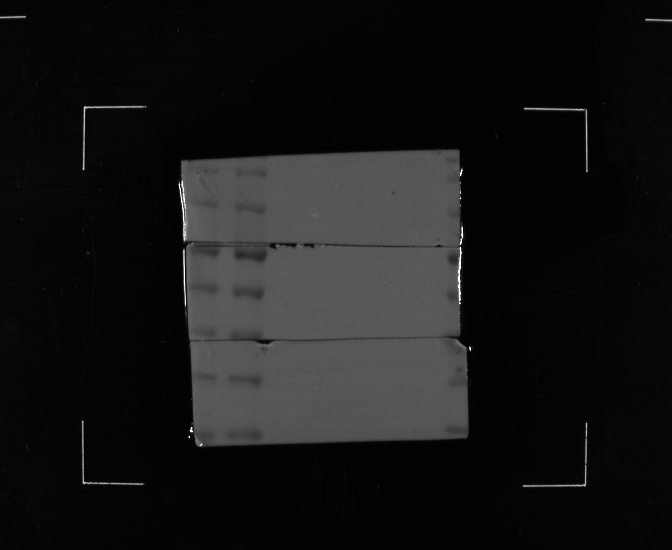

Supplement: Supplemental Information 3 [file peerj-11-16122-s003.zip › WB supplement File/First repeat/PLAUR/1í¬í¬ALL_20230519_151634_00.06.532_8bit.tif]

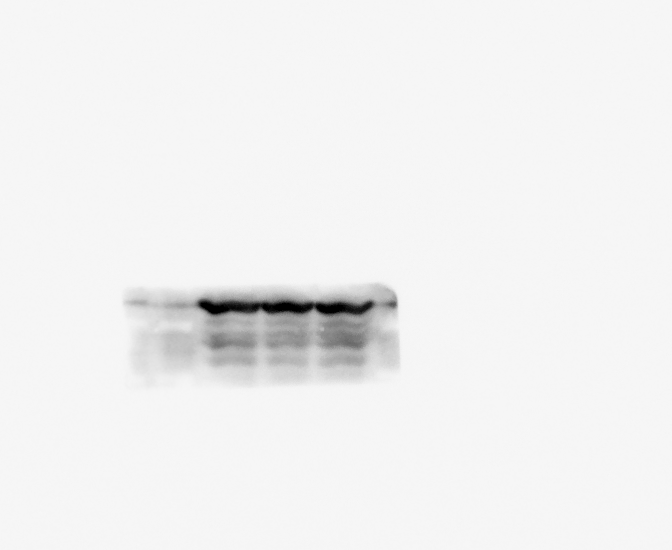

Supplement: Supplemental Information 3 [file peerj-11-16122-s003.zip › WB supplement File/First repeat/PLAUR/1í¬í¬GAP_20230519_150613_00.01.451_24bit(2).tif]

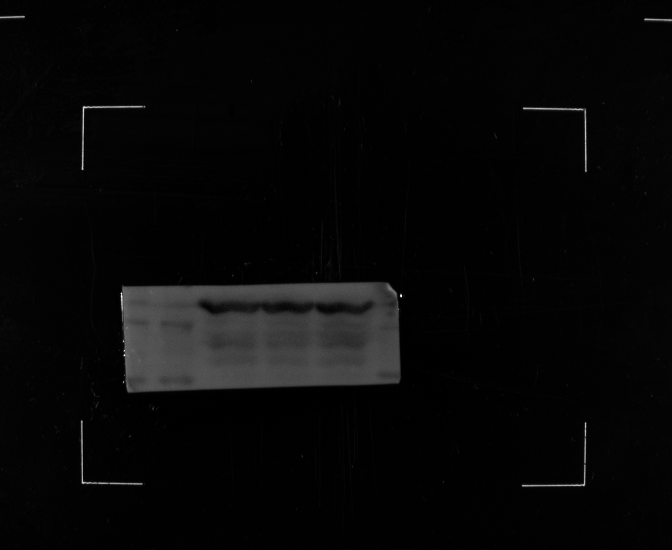

Supplement: Supplemental Information 3 [file peerj-11-16122-s003.zip › WB supplement File/First repeat/PLAUR/1í¬í¬GAP_20230519_150613_00.01.451_24bit.tif]

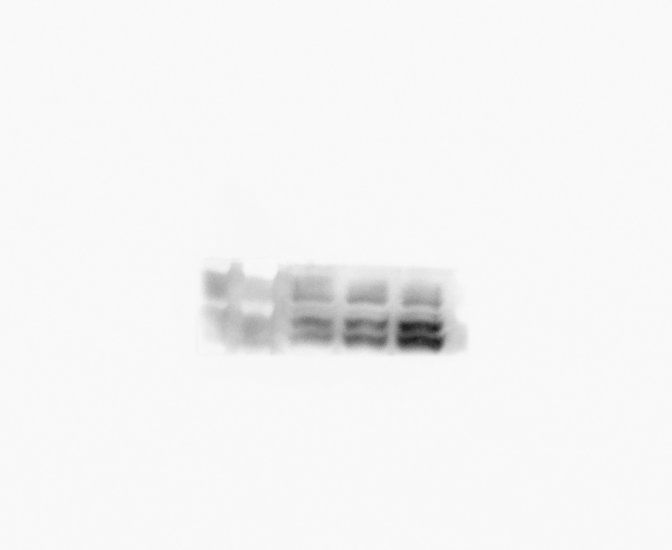

Supplement: Supplemental Information 3 [file peerj-11-16122-s003.zip › WB supplement File/First repeat/PLAUR/1í¬í¬PL_20230519_151131_00.07.638_24bit(0).tif]

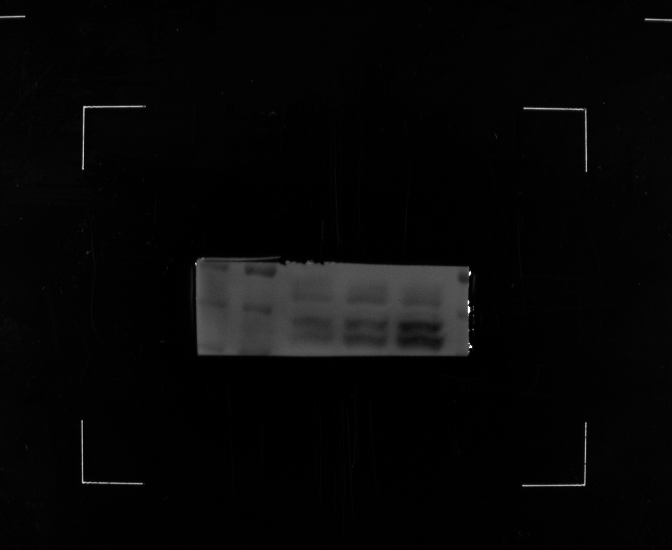

Supplement: Supplemental Information 3 [file peerj-11-16122-s003.zip › WB supplement File/First repeat/PLAUR/1í¬í¬PL_20230519_151131_00.07.638_24bit.tif]

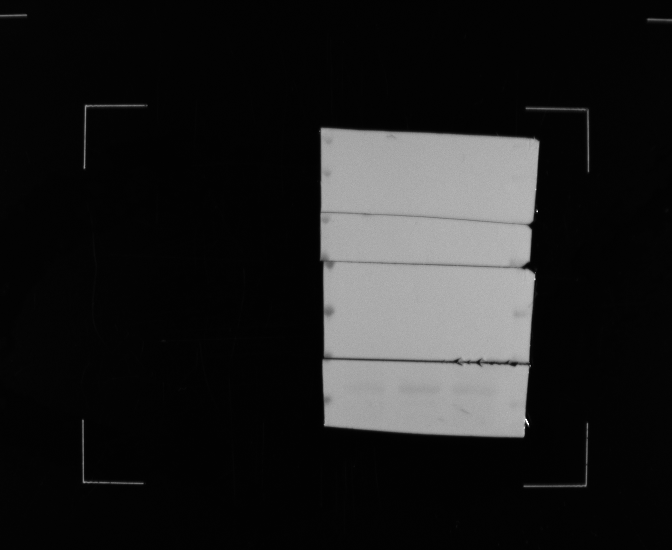

Supplement: Supplemental Information 3 [file peerj-11-16122-s003.zip › WB supplement File/Second repeat/CSF1R/Aí¬í¬2_20230509_163257_00.00.135_24bit.tif]

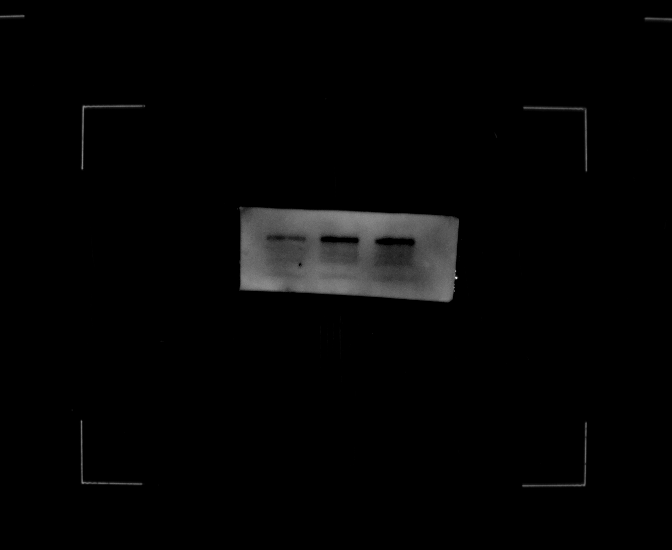

Supplement: Supplemental Information 3 [file peerj-11-16122-s003.zip › WB supplement File/Second repeat/CSF1R/Cí¬í¬2_20230509_162208_00.27.574_24bit.tif]

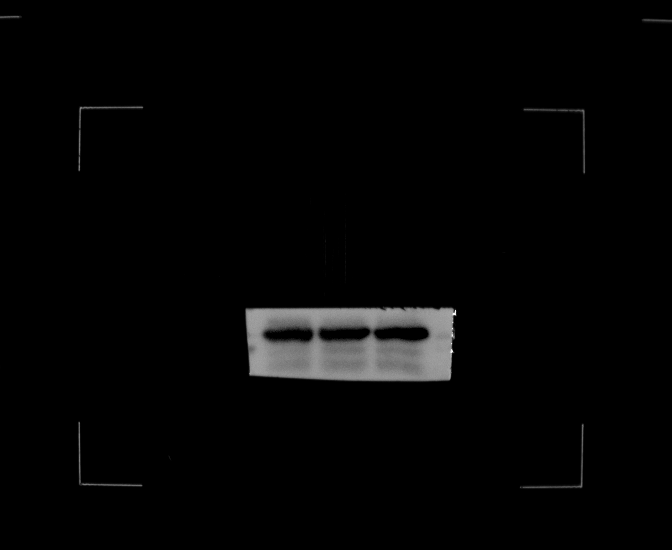

Supplement: Supplemental Information 3 [file peerj-11-16122-s003.zip › WB supplement File/Second repeat/CSF1R/Gí¬í¬2_20230509_160340_00.00.050_24bit.tif]

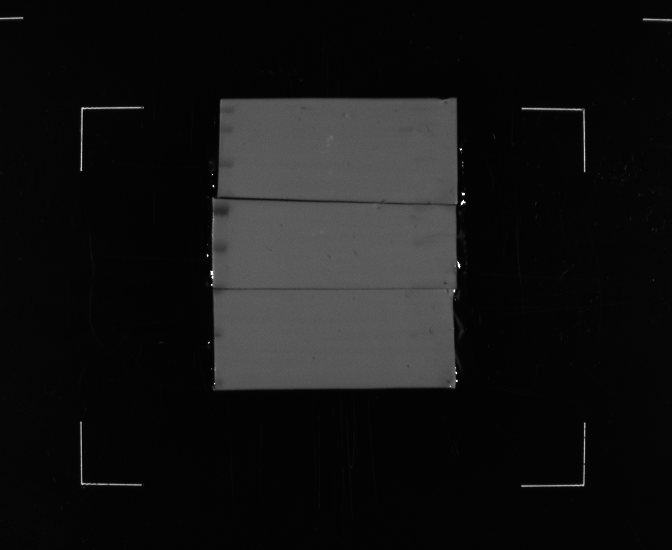

Supplement: Supplemental Information 3 [file peerj-11-16122-s003.zip › WB supplement File/Second repeat/PLAUR/2í¬í¬A_20230519_153814_00.00.952_0.tif]

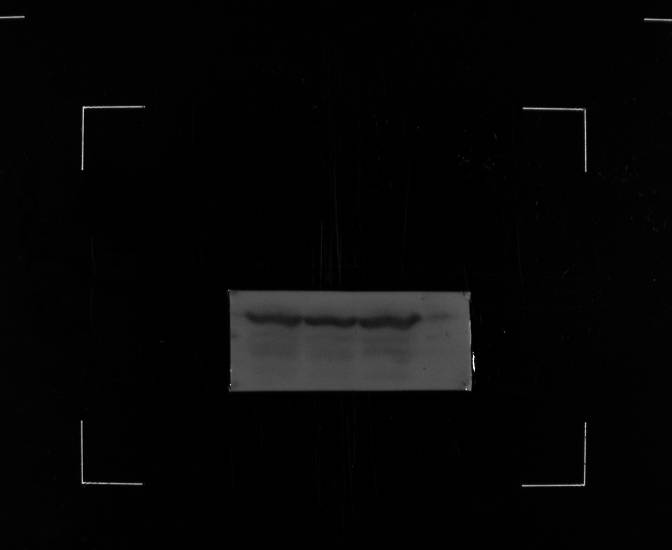

Supplement: Supplemental Information 3 [file peerj-11-16122-s003.zip › WB supplement File/Second repeat/PLAUR/2í¬í¬G_20230519_153420_00.01.115_24bit(0).tif]

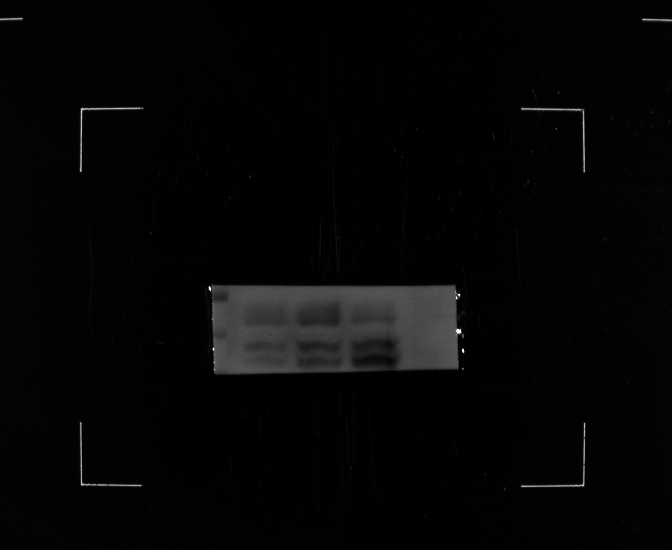

Supplement: Supplemental Information 3 [file peerj-11-16122-s003.zip › WB supplement File/Second repeat/PLAUR/2í¬í¬P_20230519_152438_00.09.748_24bit(0).tif]

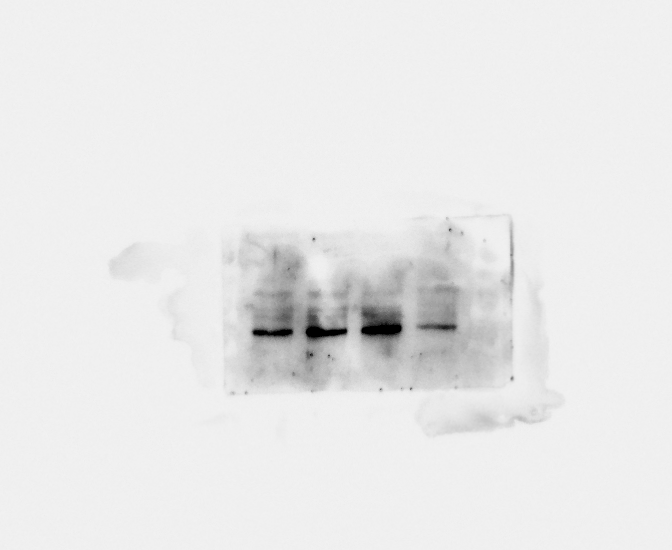

Supplement: Supplemental Information 3 [file peerj-11-16122-s003.zip › WB supplement File/Thrid repeat/CSFí¬í¬25UL_20230505_151748_00.05.515_8bit.tif]

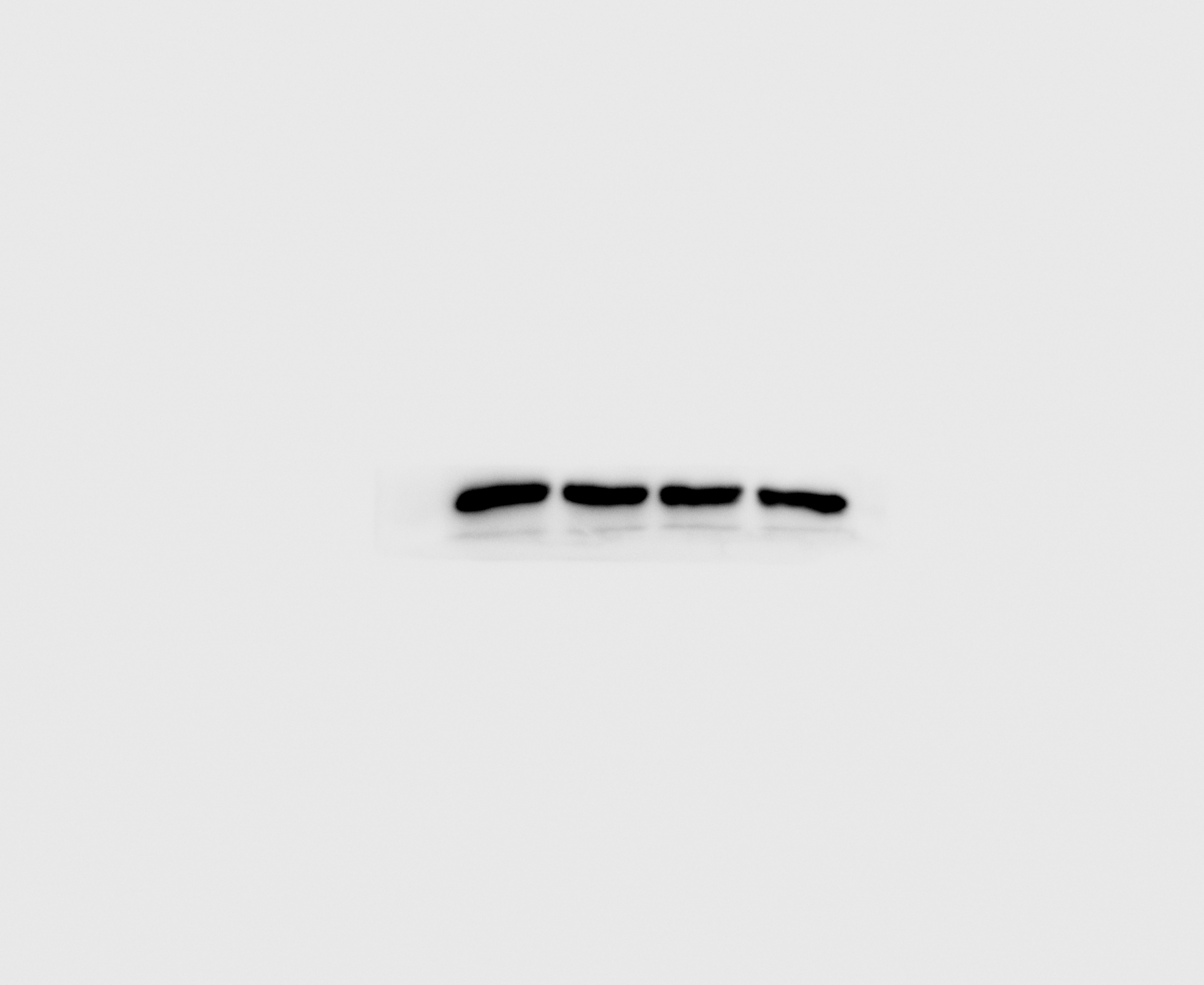

Supplement: Supplemental Information 3 [file peerj-11-16122-s003.zip › WB supplement File/Thrid repeat/GAPí¬í¬25UL_20230505_150135_00.03.344_8bit.tif]

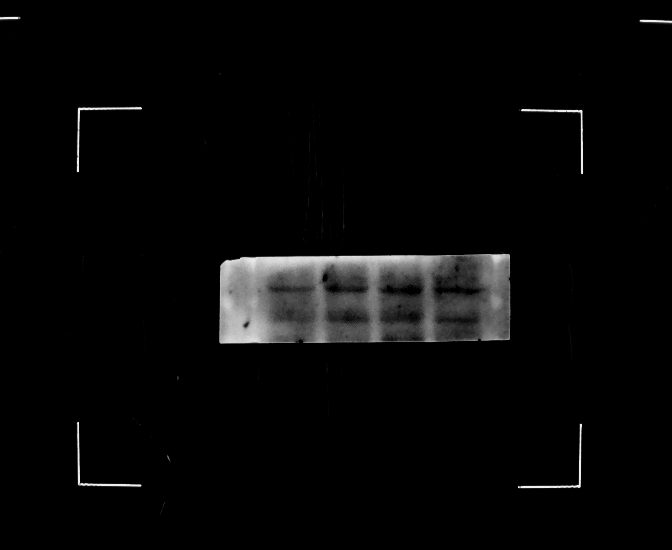

Supplement: Supplemental Information 3 [file peerj-11-16122-s003.zip › WB supplement File/Thrid repeat/PLAUí¬í¬25UL_20230505_151259_00.10.629_8bit.tif]
